# Development and Validation of a RP-HPLC Method for Determination of Cyclosporine in Capsule

**DOI:** 10.4103/0250-474X.65030

**Published:** 2010

**Authors:** F. Aziz, A. Gupta, M. F. Khan

**Affiliations:** Ranbaxy Research Laboratories, R & D 3, Gurgaon-122 001, India

**Keywords:** Cyclosporine, immunosuppressant, RP-HPLC, Validation

## Abstract

A simple, specific and accurate reverse phase high performance liquid chromatographic method was developed for the determination of cyclosporine in capsule dosage form. XTerra C18 column was used as stationary phase with mobile phase acetonitrile in combination with 0.1% trifluoro acetic acid buffer and pH is adjusted to 1.4. Method was developed in an isocratic run of 20% trifluoro acetic acid with 80% acetonitrile for 10 min, at flow rate of 1 ml/min. Effluents were monitored at 210 nm. Retention time of cyclosporine was 3.855 min. The method was validated for specificity, linearity, accuracy, precision, limit of quantification, limit of detection, robustness and solution stability. Limit of quantification and limit of detection of cyclosporine was found to be 100 ng/ml and 200 ng/ml. Recovery was found to be in the range of 98.08-101.55%. The proposed method was successfully applied for the quantitative determination of cyclosporine in a capsule dosage form.

Cyclosporine is an immunosuppressant drug used in post allogenic organ transplant to reduce the activity of patient's immune system[1]. It is a cyclic non ribosomal peptide of 11 amino acids, with molecular weight 1202.61. Its molecular formula is C_62_H_111_N_11_O_12_, and chemically designated as (R-(R*,R*-(E)))-cyclic-(L-alanyl-D-alanyl-N-methyl-L-leucyl-N-methyl-L-leucyl-N-methyl-L-valyl-3-hydroxy-N,4-dimethyl-L-2-amino-6-octenoyl-L-α-amino-butyryl-N-ethylglycyl-N-methyl-L-leucyl-L-valyl-N-methyl-L-leucyl). The important property of cyclosporine is its ability to inhibit the production of cytokines, which are involved in regulation of T-cell activation. Particularly it inhibits the synthesis of interleukin 2(IL-2) at the level of gene transcription[2]. This activity is achieved by complex formation with cyclophilin, which leads to reduced function of effector T cells[3]. A literature survey regarding quantitative analysis of cyclosporine revealed that attempts were made to develop analytical method using HPLC, LC-MS[4] and RP-HPLC. The present study was attempted to develop a method of short run time, simple solvent system, and better peak shape with good resolution.

The liquid chromatographic system consisted of HPLC-Waters Alliance LC-Module 1, containing PDA detector and Rheodyne injector with 20 µl fixed loop. Analysis performed using Empower software on XTerra C18 column (150×4.6) mm and 5 µm particle size. A Shimadzu electronic balance was used for weighing purpose. Reference standards were provided from Ranbaxy Research Laboratory, Gurgaon, India itself and capsules formulation brand name Panimun Bioral (Panacea Biotech Ltd, India) containing labeled amount of 100 mg cyclosporine, were procured from local market. Acetonitrile (ACN) HPLC grade was procured from Merck and trifluoroacetic acid (TFA) from Qualligens. TFA is used as ion pairing agent in this study, it also sharpens the peak shape and improves resolution[4–8]. HPLC grade water was obtained by double distillation and purification through milliQ water purification system.

ACN of HPLC grade was taken as mobile phase in combination with TFA buffer. To the 1000 ml of milliQ water 0.1 ml of TFA was added and its pH was adjusted to 1.4 with acetic acid. For the preparation of stock, 500 mg of standard was accurately weighed and dissolved in 20 ml of mobile phase (TFA/ACN, 1:1) and was sonicated for 5 min. Standard solution was further taken in 10 ml volumetric flask and diluted in a manner to obtain the final concentration of 50 µg/ml. Sample was prepared by cutting 5 gelatinous capsules of Panimun Bioral. In 25 ml of mobile phase, 250 mg accurately weighed capsule content was dissolved. To obtain the sample concentration of 100 µg/ml in mobile phase, 0.1 ml of the sample solution was taken in 10 ml volumetric flask and diluted up to the mark with mobile phase.

A reverse phase XTerra C_18_ column at 50° was equilibrated with mobile phase (0.1% TFA, ACN) in an isocratic run (20:80). pH was adjusted to 1.4 using acetic acid and flow rate was maintained at 1 ml/min. Total run time was 10 min and effluents were monitored at 210 nm. Sample was injected using 20 µl fixed loop.

Appropriate aliquots of cyclosporine stock solutions were taken in different 10 ml volumetric flasks and diluted up to the mark with mobile phase to obtain the final concentration of 5, 10, 15, 20, 25 µg/ml. Duplicate injections using 20 µl fixed loop were made and chromatograms were recorded at 210 nm. Quantification was carried out by keeping peak area and concentration of drug to straight line equation and it was found linear with correlation coefficient (r)=1. The method was validated for accuracy, limit of quantitation and robustness.

Accuracy of the method was determined by calculating recovery of cyclosporine by method of standard addition. Known amount of cyclosporine (10, 15, 20 µg/ml) were added to prequantified sample solutions and the amount was estimated by measuring the peak area ratios by fitting these values to the straight line equation of calibration curve.

The intraday and interday precision study was carried out by estimating the responses 3 times on same day and 3 times on different days for 3 different concentrations (10, 15, 20 µg/ml) and the results are reported in terms of relative standard deviation (RSD) in Table 1.

**TABLE 1:** SUMMARY OF VALIDATION PARAMETERS

Parameters	CYSP
Detection limit (µg/ml)	0.100
Quantitation limit (µg/ml)	0.200
Accuracy (%)	99.38-101.11%
Precision (RSD %)	
Intraday (n=3)	0.95-1.85
Interday (n=3)	1.25-3.98
Repeatability (RSD, n=3)	0.35-0.43

RSD indicates relative standard deviation.

Repeatability studies were carried out by estimating response of 3 different concentrations of cyclosporine (10, 15, 20 µg/ml) for 3 replicate determinations and results are reported in terms of relative standard deviation (RSD) in Table 1.

Specificity of above described RP-HPLC method was determined by various parameters like retention time, resolution and tailing factor[9–11]. In the present study tailing factor of the peak was 1, with satisfactory resolution. Retention time and standard deviation (±SD) was found to be 3.58 minutes and ±0.53364 for six replicates. Thus, the peak obtained was sharp and have clear baseline.

A calibration curve was prepared using concentrations in the range of 0.100-0.200 µg/ml. Standard deviation of y-intercepts of regression lines were determined and kept in the following equation for the determination of detection limit and quantitation limit. Detection limit=3.3 σ/s, quantitation limit=10 σ/s, where σ is the standard deviation of y-intercepts of regression lines and s is the slope of the calibration curve.

Robustness of the method was studied by changing the composition of organic phase by ±5% and the pH by ±0.02%, and also by observing the stability of the drug for 24 h at 35° temperatures in mobile phase. In order to check the stability, both the standard and sample solutions were analyzed over a period of 8 h at room temperature. The results for both the solutions remained almost unchanged and no significant degradation was observed within the indicated period.

Optimization of mobile phase was performed based on resolution, asymmetric factor and peak area obtained. Different mobile phase were tried but well resolved and good symmetrical peaks were obtained with mobile phase TFA and ACN (20:80, pH 1.4). Retention time of cyclosporine was found to be 3.855 min with satisfactory resolution. Overlain UV spectra of cyclosporine showed that drug absorbs appreciably 210 nm, so this wavelength was selected as the detection wavelength in liquid chromatography. Calibration curve for cyclosporine was obtained by plotting peak area versus concentration over the range of 5 µg/ml, and it was found to be linear with correlation coefficient (r)=1.

The detection limit and quantitation limit for cyclosporine was 0.100 µg/ml and 0.200 µg/ml, which suggest that nanogram quantity of it can be estimated accurately. The validation parameters are summarized in Table 1. The recovery of cyclosporine was found to be 99.38-101.11%. The proposed method was met for cyclosporine with the parameters of system suitability test as retention time 3.8 min with resolution 1.8 and tailing factor 1.

The present study described a new RP-HPLC method using simple mobile phase for the estimation of cyclosporine in oral dosage form. The method was validated and was found to be simple, sensitive, accurate and precise.
